# 

**Published:** 1869-04

**Authors:** 


### BOOK NOTICES.

THE MERCHANTS AND BANKERS' ALMANAC FOR 1869, containing list of 1,650 National Banks; 300 State Banks; 1,400 Private Bankers in the U. S.; Banks and Bankers in Canada; 1,200 Bankers and Brokers in New York city, including names of members of the New York Stock Exchange, the Open Board of Brokers, the Gold Board and the Mining

Board; the daily price of gold at New York, 1863 to 1868; Alphabetical List of 2,000 Cashiers; Engravings of New Bank Buildings, etc. [The names of New Banking Houses inserted in "Bankers' Magazine" monthly, and "Bankers' Almanac," annually, without charge.] Address, "Bankers' Magazine, P. O. Box 4574, New York."

FISK'S PORTABLE WRITING BOARD.—One of the best articles that we have ever seen for the use of Reporters, Clerks, Book-keepers, Business or Professional men. For out-door or field reporting of races, ball matches or anything of the kind, nothing could be better, as it can be carried about in the hand, while the paper remains securely in its place. For letter writers it is *extremely useful*, as it offers not the *ghost of a chance* for making the oft-repeated mistake of putting a letter into the wrong envelope. Every man who uses pen, pencil or paper, can ill afford to be without one of them. The public will be sure to appreciate it, as it is invaluable.—*Springfield Union.*

It is certainly a very convenient article.—*Hall's Journal of Health.*

CRAIG MICROSCOPE, ETC.—Testimonial in favor of the articles for sale by John Hall, Bergen, Hudson Co., N. Jersey.

WASHINGTON, D. C., Jan. 20th, 1868.

JOHN HALL, ESQ.—*Dear Sir:*—I have just set aside the Craig Microscope which I purchased of you several years since. I have been examining some Cancer-cells, and the result is so satisfactory that I am now doing what I have desired to do many times before when using this little instrument, and that is, to express to you my gratitude for directing my attention to it. I keep it on my desk all the time, and find it invaluable. It very satisfactorily determines the character of the Blood-globules, Milk-globules, Pus, and the various Deposits of Urine. It is, in fact, indispensable; and I only regret that I did not have it twenty years before.

Very respectfully, S. L. LOOMIS, M. D.,
Professor of Chemistry and Toxicology, Medical Faculty,
Georgetown College, D. C.

Four Pillars of Temperance, by John W. Kirston. Published by the National Temperance Society and Publication House, 172 William street, New York. 1869. A 16mo. of 240 pp. Price 75 cents. The four pillars are Reason, Science, Scripture and Experience. It is well written, useful and instructive. The sections on the laws of physiology, the laws of health and life, is alcohol food, does it help digestion, &c., &c., are well stated, and are presented with great force.

"Library of Education." J. W. Schermerhorn & Co., publishers, 14 Bond Street, New York. 194 pp. 24 mo. Price 15c., or 20c. prepaid to any part of the country. This library is to be selected from the best authors of all countries, the first vol. being "Sound Thoughts concerning Education, by John Locke." Among the names of forthcoming volumes will be Bacon, Milton, J. Stuart Mill, Carlysle, Prof. De Morgan Sir Edmund Head, &c., &c. It is an admirable idea, and we hope the publishers will carry it out with judgment and fidelity to the great public of readers.

Report of the Pennsylvania Hospital for the Insane, for the year 1868. By Thomas S. Kirkbride, M.D., Physician-in-Chief and Superintendent. This is considered the most successfully conducted Insane Asylum in the United States, owing to the fidelity and ability of Dr. Kirkbride.

"The Dental Cosmos." Philadelphia. $2.50 a year. For February is a very valuable number; every respectable and educated dentist owes it to himself and his patrons to acquaint himself with the progress of mental science, of which the *Cosmos* gives a faithful monthly record.

"The Galaxy." $4 a year. 500 Broadway, New York. Makes its March appearance; it, with this Journal, is sent for one year for $4. The article, "Tea and its Adulterations," by Prof. John C. Draper, M. D., 8 clearly printed pages, conveys an amount of practical, reliable and useful observations of the nature, uses and properties of tea, which merits the careful perusal of every person of intelligence in the land. Send forty cents for the March number as a specimen, to the *Galaxy*, 500 Broadway, New York.

